# Supplementary material for: Mixed-mode in-memory computing: towards high-performance logic processing in a memristive crossbar array
Source: Commun Eng. 2025 Sep 24;4:163. doi: 10.1038/s44172-025-00461-y (PMC12460548; doi:10.1038/s44172-025-00461-y)
Supplement: Supplementary file 2 — Supplementary Information [file 44172_2025_461_MOESM2_ESM.pdf]

Mixed-Mode In-Memory Computing: Towards High-Performance Logic Processing in A Memristive Crossbar Array

Nan Du[1,2*], Ilia Polian[3], Christopher Bengel [4, †], Kefeng Li[1,2], Ziang Chen[1,2], Xianyue Zhao[1,2], Uwe Hübner[1], Li-Wei Chen[3], Feng Liu[5], Massimiliano Di Ventra[6], Stephan Menzel[5], Heidemarie Krüger[1,2]

[1]Leibniz Institute of Photonic Technology (IPHT), Albert-Einstein-Str. 9, Jena, 07745, Germany.
[2]Institute for Solid State Physics, Friedrich Schiller University Jena, Helmholtzweg 3, Jena, 07743, Germany.
[3]Institute of Computer Engineering and Computer Architecture, University of Stuttgart, Pfaffenwaldring 47, Stuttgart, 70569, Germany.
[4]Institute of Materials in Electrical Engineering and Information Technology, RWTH Aachen University, Sommerfeldstraße 18, Aachen, 52074, Germany.
[5]Peter Grünberg Institut (PGI-7), Forschungszentrum Jülich, Wilhelm-Johnen-Straße, Jülich, 52428, Germany.
[6]Department of Physics, University of California, San Diego, 9500 Gilman Drive, La Jolla, CA 92093-0319, USA.

*Corresponding author(s). E-mail(s): nan.du@leibniz-ipht.de;

† Christopher Bengel works now at HELLA GmbH & Co. KGaA — Rixbecker Strasse 75, 59552 Lippstadt, Germany.

SUPPLEMENTARY INFORMATION

A Classification of representative memristive logic designs

The intrinsic functional behavior of memristors, characterized by voltage-triggered latching effects and memristance-based memorization, sets them apart from their CMOS counterparts. The stored memristance (M) and the applied voltages (V) at their terminals act as both logical inputs and outputs, facilitating their use in logic processing. Nanoscale memristive devices harness these unique properties to perform logic operations, offering a promising approach for in-memory computing paradigms where logic processing and computation occur directly within the memory block. As illustrated in Fig. S1, the memristive logic designs presented in the literature can be systematically classified based on distinct logic kernels: MI, MO, VI, and VO. These designs are structured as combinations of one input kernel (MI or VI), representing the logic input variable, and one output kernel (MO or VO), representing the output logic variable. Through this categorization, we analyze the strengths and limitations of these designs, offering valuable insights into their compatibility with specific logic kernels.

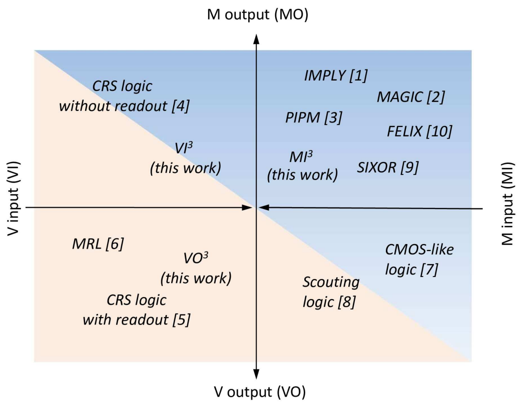

Fig. S1 Landmap of representative memristive logic designs distributed in logic kernels MI, MO, VI and VO.

Table S1 Summary of representative memristive logic designs in comparison to 3-input MI^3, VI^3 and VO^3 logic operations.

Logic design	Logic inputs	Logic outputs	Logic functions	Computation style	# devices †	# cycles	Gate error rate
IMPLY [1]	MI 2-input	MO	IMPLY	Sequential	2M+1R	1	High
MAGIC [2]	MI 2-input	MO	e.g.NOR, NAND	Sequential	3M	1	High
PIPM [3]	MI 2-input	MO	e.g.NOR, XNOR	Parallel	2M	1	Low
CRS logic without readout [4]	VI 2-input	MO	14 gates except XOR/XNOR	Sequential	1M	1	Very low
CRS logic with readout [5]	VI 2-input	VO (2-input VI)	16 gates possible	Sequential	1M	2	Very low
MRL [6]	VI 2-input	VO	AND, OR	Parallel	2M	1	Low
CMOS-like logic [7]	MI 2-input	VO	NOR, NAND	Sequential	4M	1	Low
Scouting logic [8]	MI 2-input	VO	AND, OR, XOR	Sequential	2M+2T	1	Very low
SIXOR [9]	MI 2-input	MO	XOR	Sequential	5M+1T	1	Very high
FELIX [10]	MI n-input	MO	NOR,OR	Sequential	nM	1	Very high
MI^3 (this work)	MI 3-input	MO	-	Sequential	3M	1	High
VI^3 (this work)	MI(/VI) 3-input	MO	-	Sequential	1M	1	Very low
VO^3 (this work)	VO 3-input	MO	-	Sequential	3M	1	Very low

† In the column labeled "# of devices", M represents memristor cells, and R represents resistor cells.

For instance, the representative memristive logic designs such as Material Implication Logic (IMPLY) [1], Memristor-Aided Logic (MAGIC) [2], and Fast and Energy-Efficient Logic in Memory (FELIX) [10] exemplify the fusion of MI and MO kernels, wherein both inputs and outputs are represented by nonvolatile memristive states M, also known as the stateful logic concept. For instance, the MAGIC logic design illustrated in Fig. S2a, comprises at least two input memristive cells (M_1/M_2) and one output cell (M_3). When voltages (V_{in1} and V_{in2}) are applied, an input-dependent resistive voltage divider forms between the inputs and the output. Under specific input configurations, the output cell selectively switches its state if the voltage drop (V_{com}) exceeds a certain threshold. Such logic designs often achieve universal gate types, storing the output permanently within the crossbar array upon generation. Note that, in this work, "universality" refers to the capability of one logic kernel to realize arbitrary functions using the corresponding logic designs in a cascading manner by using the logic inputs $\{\text{const-0}, \text{const-1}, x_1, \overline{x}_1, x_2, \overline{x}_2, \ldots\}$. For instance, considering one logic design within the VI kernel—specifically the CRS logic design [4] without readout allowed—to realize 3-input logic functions through cascading operations (sequential writing without current sensing) can achieve only 104 out of the 256

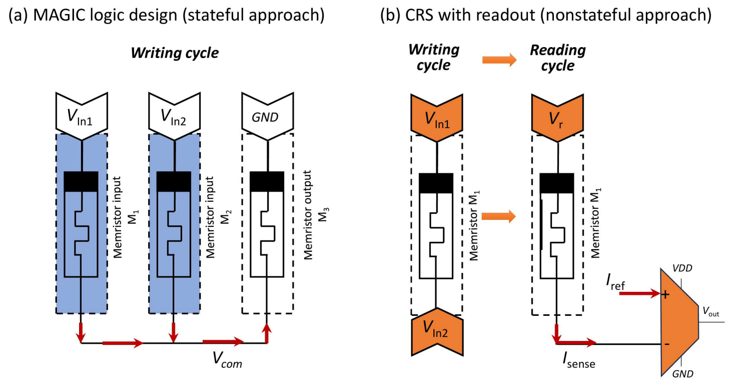

Fig. S2 Comparative illustration between (a) stateful MAGIC [2] and (b) nonstateful CRS [5] logic designs, where the logic input variables are marked in blue and orange, respectively.

possible functions (2^8), demonstrating that the VI kernel is not universal. This minimizes the need for additional memory operations and facilitates the straightforward reuse of the logic output for subsequent operations. Nonetheless, these designs face following limitations:

- Prolonged operation sequences during logic processing are common due to the restricted number of gate types applicable exploiting specific memristive technologies. For instance, in Ref. [11], the RESET process (LRS $\rightarrow$ HRS) is implemented in the output cell of the MAGIC logic design to achieve two-input Boolean NOR/-NAND gates. In comparison, Ref. [12] demonstrates the implementation of the SET process (HRS $\rightarrow$ LRS) in the output cell to realize NOT-material Implication (NIMP), OR, and NOT gate types.
- Practical logic cascading may encounter an elevated error rate of up to 70% [13, 14], attributed to stochastic variability inherent in nanoscale memory devices, as experimentally analyzed in published works. This high error rate renders large-scale logic processing impractical.
- Voltage-divider circuit designs introduce additional uncertainty in resulted memristive states of input and output cells (see details in descriptions in Supplementary Information C).

- Recent investigations underscore the necessity for more than three input memristive cells within a single stateful logic gate to achieve higher computing efficiency. This requirement is evidenced by designs such as the single-cycle in-memristor XOR (SIXOR) [9] and FELIX logic [10]. However, we contend that a stateful approach incorporating more than three cells is not practically feasible due to the heightened error rates observed experimentally even with just three cells. Including multiple devices within a single logic operation would significantly elevate the error rates.

These issues exacerbate the error rate during cascading of such stateful logic gates. In our study, we introduce the universal MI^3 logic operation, which represents an expanded iteration of the MAGIC logic operation. This advancement not only broadens the input variables from 2 to 3, facilitating the cascading process between MI and VI kernels as well as within the MI kernel (see detailed discussions at the end of this section), but also addresses significant challenges such as input drift and partial switching issues within input and output cells (see detailed discussions in Supplementary Information C), by flexibly combining logic operations in VI kernel before and after MI^3. It is noteworthy that the goal of our work is not to resolve all limitations of individual kernel but to leverage the complementary strengths of MI and VI kernels to address the shortcomings of each. By combining the universal capabilities of MI^3 with the robustness and bidirectional transition flexibility of VI^3 logic designs, the mixed-mode approach achieves enhanced resilience, reduced processing cycles, and the elimination of VO readout, enabling universal and more reliable logic processing.

The representative logic designs such as complementary resistive switching (CRS) logic with readout [5] exemplifies the fusion of VI and VO kernels, where two subsequent operational steps are required (also known as one type of non-stateful logic concepts). As illustrated in Table S1, CRS logic with readout employing a single memristive cell (M_1) as input and output cell. Input-dependent writing and reading voltage values are applied to both cell terminals in both writing and reading cycles. Notably, the logic inputs are delineated by voltage applied to the device terminals (marked in orange in Fig. S2). In the writing cycle, upon applying specific input combinations, which generate a voltage difference between the terminals, the cell undergoes a deterministic switch to either LRS or HRS through the SET/RESET process. Otherwise, it remains in its initial stored state. The output is permanently stored within the crossbar array directly as it is computed. To acquire the output, expressed either as current or voltage sensed from the memristive cell, a reading cycle with readout operation in VO kernel is mandated. This logic design facilitates deterministic switching processes on a single device and demonstrates resilience to device variations. Additionally, it entails significantly reduced area and latency costs compared to its stateful counterparts, as a diverse range of logic gate types can be achieved using just one cell. Nonetheless, these designs face following limitations:

- Such a logic design without a readout process can not realize arbitrary functions in a cascading manner by using the input variables of the function to be computed (literals) $\{\text{const-0}, \text{const-1}, x_1, \overline{x}_1, x_2, \overline{x}_2, \ldots\}$. For example, the CRS logic [4], exemplifies the integration of VI and MO kernels without readout VO kernel. In CRS logic, voltage applied to device terminals serves as inputs, while output is represented

through nonvolatile memristive states M (also known as one type of nonstateful logic concepts). Notably, in the work in Ref. [4], it demonstrated that CRS logic without readout can implement 14 types of logic gates (excluding XOR and XNOR) using a single cell. However, using such a logic design to implement 3-input logic functions in a cascading manner (without additional initialization) can realize only 104 out of the 256 possible arbitrary functions.

- During logic processing, in both writing and reading cycles, data for logic operations or cascading must be accessed by peripherals to be applied as voltage values to the memristor's two terminals. This necessitates repeated usage of the readout VO kernel, requiring peripheral circuitry and introducing additional latency and power cost. Further insights into the readout VO kernel are elaborated in Supplementary Information B.

In this work, we propose a 3-input VI^3 logic operation, representing an extended iteration of CRS logic (without readout). Unlike conventional CRS logic design, this approach expands the scope of input variables from 2 to 3 and establishes a complete gate set through integration with MI^3 logic operation, thereby eliminating the need for a readout operation. This elimination of the readout operation during logic cascading is a significant advantage of our work, which will be detailed further in the next section (in Supplementary Information C). Besides that, the 3-input logic operations proposed in this work offer several distinct advantages: Firstly, they embody a synergistic design by integrating two types of 2-input gates, such as the $\overline{p} \cdot q$ and $\overline{p} + q$ gates (p and q are logic input variables in 2-input logic gates) from the CRS logic family, into a single 3-input operation. This integration reduces circuit-level design complexity and enables faster processing per cycle. Additionally, the 3-input configuration fully exploits the dual physical properties of memristors—stored resistance and applied electrode voltage—by using both resistance states and operating voltages as input variables. This approach achieves efficient and accurate logic operations without requiring additional readout logic, significantly improving overall performance. Secondly, the 3-input design provides substantial efficiency gains in logic cascading. Unlike traditional 2-input gates that require memristive devices to be re-initialized before each operation, the 3-input design allows the output from one operation to be immediately reused as input in subsequent operations, bypassing re-initialization steps. This direct integration enhances throughput, reduces the number of sequential stages, and supports a streamlined and efficient design flow, ensuring smooth operation between MI and VI kernels. Furthermore, the 3-input configuration simplifies the implementation of complex logic functions within automation tools by reducing the need for additional stages and design complexity. This makes it a practical solution for managing memristive systems in crossbar arrays, improving both automation tool development and overall circuit design. The last but not the least, our work adopts a holistic co-design methodology that integrates device, circuit, and system-level considerations to maximize performance. By addressing these levels in a coordinated manner, the approach leverages interdependencies that enhance system capabilities. The 3-input logic design optimizes automation tool development, which in turn facilitates the effective implementation of the design within crossbar architectures, achieving a high degree of efficiency, flexibility, and functionality.

The Boolean expressions that represent the operations of both the MI^3 and VI^3 logic operations are described as follows: the Boolean function for the MI^3 operation is

$m_{y_1} = (m_{x_1} \cdot \overline{m}_{x_2} \cdot \overline{m}_{x_3})$,

while the Boolean function for the VI^3 operation is as

$m_{y_1} = (\overline{m}_{x_1} \cdot v_{x_2} \cdot \overline{v}_{x_3}) + (m_{x_1} \cdot \overline{v}_{x_2} \cdot v_{x_3}) + (m_{x_1} \cdot v_{x_2} \cdot \overline{v}_{x_3}) + (m_{x_1} \cdot v_{x_2} \cdot v_{x_3})$.

While simpler logic functions like XOR or XNOR can also be implemented, such examples do not fully demonstrate the strengths of our mixed-mode approach in terms of cycle efficiency and reduced device usage. In this work, we experimentally demonstrate the capabilities of our system by implementing not only a full adder but also computationally intensive functions such as the 4-bit Sbox. The 4-bit Sbox, with its high nonlinearity and resistance to cryptographic attacks, serves as a challenging benchmark to highlight the robust capabilities and optimized performance of our system in managing real-world, complex computational tasks.

B Readout VO kernel and its limitations

The readout VO kernel is implemented through one readout logic step, necessitating the sensing of memristance state stored in the cell by applying input-dependent reading voltage values across the two terminals of the memristor. If VO kernel is allowed, the current or voltage values sensed from memristive cells are used as logic output or input variables in the cascaded logic gates.

Some devices support a rich readout behavior. Fig. S3 demonstrates the readout logic determined in $BiFeO_3$ memristor series, including $BiFeTiO_3$, $BiFeO_3$ and $BiFeTiO_3/BiFeO_3$ memristive devices. The fabrication process of experimental $BiFeO_3$ memristor series is described in Experimental Section. The experimentally tested $I-V$ characteristics of $BiFeTiO_3$, $BiFeO_3$ and $BiFeTiO_3/BiFeO_3$ memristive devices, recorded under ramping pulses with voltage step of 0.1 V and step time of 0.1 s are shown in Fig. S3a, Fig. S3b, and Fig. S3c, respectively. The arrows indicate the sweeping direction of the applied ramping voltage values, which are applied to the respective top electrodes. The $BiFeO_3$ memristor series are demonstrating bipolar resistive switching behaviors, while possessing different hysteretic switching dynamics. For example, the hysteretic behavior can be found in negative or positive bias range in $BiFeTiO_3$ (Fig. S3a) or $BiFeO_3$ (Fig. S3b) memristive devices, respectively, due to the constructed rectifying/nonrectifying contact with flexible Schottky-like barrier height near to top electrode or bottom electrode interfaces [15]. The deposition of a $BiFeTiO_3$ film followed by a $BiFeO_3$ film on Pt/Ti substrate defines the $BiFeTiO_3/BiFeO_3$ bilayer structure as illustrated schematically in the inset of Fig. S3c. Flexible barriers formed at both top and bottom interfaces for the bilayer structure results in hysteretic behaviors both in the negative and positive bias range in $BiFeTiO_3/BiFeO_3$ memristive device [5]. In $BiFeO_3$ memristor series, the positive low resistance state (PLRS) and negative high resistance state (NHRS) are attainable upon an application of a writing pulse with positive amplitude in $BiFeO_3$ and $BiFeTiO_3/BiFeO_3$ devices, while the negative low resistance state (NLRS) and positive high resistance state (PHRS) can be recorded after applying a negative writing pulse in $BiFeTiO_3$ and $BiFeTiO_3/BiFeO_3$ devices. Therefore, in $BiFeO_3$ memristor series, it is possible to readout the PLRS and

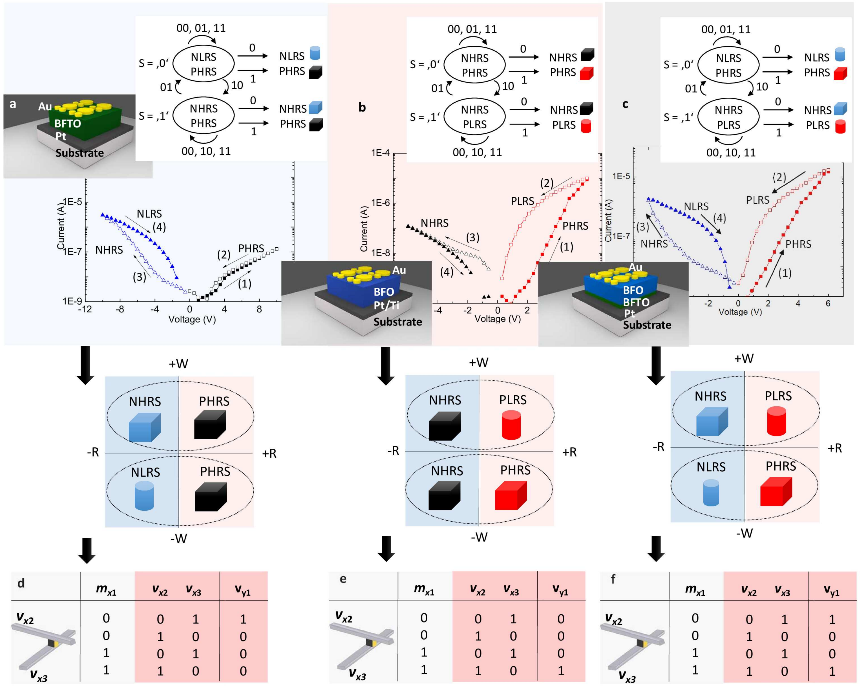

Fig. S3 Demonstration of readout VO^3 logic operations in $BiFeO_3$ memristor series. For (a) $BiFeTiO_3$, (b) $BiFeO_3$ and (c) $BiFeTiO_3/BiFeO_3$ memristive devices, the device schematics, $I-V$ characteristics, logic related switching dynamics, and the state definitions according to the polarity of writing and reading biases are demonstrated (Cylinders: LRS, Cubes: HRS. Red colored shapes represent reconfigurable states in positive bias range, while blue colored ones in negative bias range. Non-reconfigurable states are presented by black colored shapes). The truth tables of technology dependent readout logic VO^3 are determined for (d) $BiFeTiO_3$, (e) $BiFeO_3$ and (f) $BiFeTiO_3/BiFeO_3$ memristive devices. Note that the state S in (a) corresponds to m_{x_1} in (d-f), indicating the physical configuration of the memristor after a write pulse is applied.

PHRS in a non-destructive manner by a positive reading bias of 2 V to top electrode and grounding bottom electrode, while attaining the NLRS and NHRS by a positive reading bias of 2 V to bottom electrode and grounding top electrode. The performance metrics, including retention, endurance, switching ratio, and variations of the $BiFeO_3$ memristor and its variants, have been comprehensively analyzed and published in our previous works [16–20].

We determine the 3-input VO^3 logic operation, which can be in principle integrated with VI^3 and MI^3 logic operations in mixed-mode computing paradigm. The readout VO kernel is strongly technology dependent, and the 3-input VO^3 logic functions enabled by $BiFeTiO_3$, $BiFeO_3$ and $BiFeTiO_3/BiFeO_3$ memristors are shown in Fig. S3d, Fig. S3e and Fig. S3f, respectively. For instance, each type of $BiFeO_3$ memristor supports "Reading 0 ($v_{x_2} = \bar{v}_{x_3} = 0$)" and "Reading 1 ($v_{x_2} = \bar{v}_{x_3} = 1$)" operations, which return the memristance states in the negative and in the positive bias ranges,

respectively. Combining VI and VO kernel for realizing logic functions presents advantages compared to combining VI and MI, including further reduction of cycle count with the use of readout VO kernel, owing to its ability to implement various logic gate types. However, several critical considerations must be addressed when exploiting the VO kernel, particularly in comparison to MI:

- Needs of Sense Amplifiers: Readout VO kernel often involves sensing the small changes in resistance in the memristive cells. Sense amplifiers, positioned at each BL within the crossbar structure of peripheral circuitry, are commonly employed for this purpose. Tailored to the specific sensing resolutions demanded by various applications, diverse designs of sensing amplifiers are formulated featuring huge difference in power consumption. For instance, to achieve lower resolution sensing, a reference current in the μA range is employed in the sensing amplifier, discerning between '1' or '0' states by comparing it with the current from the memristor, typically operating between the nA in HRS and mA in LRS range. Conversely, for a required high resolution sensing, these amplifiers need to provide enough gain to accurately detect the resistance states in both states, which typically requires much higher power consumption for high accuracy.
- Requirement for Output-Dependent Control Logic: The logic output, derived from the current sensed by the sense amplifier, is utilized to trigger the sourcing block. For instance, a logic '1' triggers the application of the writing voltage, while a logic '0' triggers grounding to the electrode. This output-dependent control logic block sources the input required in VI kernel based on the sensed output from VO kernel during logic cascading, thereby incurring additional power consumption and latency.
- Read-Write Operations: In memory devices featuring destructive readout, performing read operations in memristive cells may disturb the stored data, necessitating subsequent write operations to restore the original state. This additional read-write cycle leads to heightened power consumption. However, harnessing such destructive reading in logic design has the potential to decrease the initialization cycles needed.

As discussed, readout VO kernel can indeed consume considerable power and latency, especially in scenarios requiring high precision, speed, and reliability. Efforts are ongoing to optimize circuit designs, materials, and algorithms to mitigate these power challenges. It is essential to highlight that within our mixed-mode computing approach, we strategically utilize the readout operations in VO kernel at the beginning of logical processing. This facilitates the retrieval of input data from memory to peripherals, thereby enabling the VI kernel. Simultaneously, this approach eliminates the need for data copying or transmission operations, ensuring that computations can be performed efficiently at any memory location without necessitating subsequent storage adjustments. Despite in this work we intentionally avoid utilizing the VO kernel during logical cascading to streamline computational processes, it is worthy to mention that VO kernel can play a significant role in intermediate stages, particularly when MI^3 operations are cascaded, and the input memristors for subsequent stages are not physically co-located with the output memristors of prior stages. In such scenarios, the VO kernel can facilitate efficient data transfer between disjoint locations, reducing the need for additional copying and transmission cycles. For instance, the use

of the VO kernel during logic processing is demonstrated in Supporting Information G. We recognize the potential of the VO kernel to relay memristance values effectively across crossbars in these situations, and further exploration of this capability is a key area planned for future work.

C Unreliable logic processing in MI kernel

Notwithstanding their fascinating functionalities, the nanoscale memory devices are inherently subject to stochastic variability [11, 21]. Originated from their stochastic nature of the switching processes, such variabilities are emerging as major issues in practical operation, and become problematic especially in practical logic cascading in large-scale logic processing. It becomes nearly fatal in stateful MI logic kernel, when multiple cells have to be accessed simultaneously while the voltage divider effect is determined by the common node voltage among the cells. The typical error rates in different cascaded stateful logic operations are evaluated, which are ranging from 54% to 92.8% as reported in Ref. [13]. Most publications about state-of-the-art logic gates and their cascading are assuming an ideal operation and do not account for such errors [22–25], which is highly unrealistic and impractical. Only few efforts are made so far to perform error correction [13, 14] by adopting the auxiliary peripheral CMOS-based circuits beside the memory array, which unfortunately deteriorate the computing efficiency and significantly sacrifice the advantages in stateful operations.

In this study, we select the self-rectifying $BiFeO_3$ memristor as our case study. Apart from their high density in passive array, which eliminates the necessity for a transistor beneath each memristor, $BiFeO_3$ memristors offer rich dynamical behavior within $BiFeO_3$ series, making them a valuable point of comparison across various devices and logic kernels. A primary motivation for choosing the self-rectifying $BiFeO_3$ memristor lies in its minimal device variations, in terms of cell-to-cell (C2C) and device-to-device (D2D) variations, in contrast to filamentary switching memristive cells. These low variations inherently mitigate the elevated error rates associated with device discrepancies.

In this work, we propose MI^3 logic operation in MI kernel utilizing $BiFeO_3$ memristors enables the execution of stateful logic concept to realize logic functions by using 3 parallel connected cells in one WL/BL within single one cycle, and directly storing logic variables as memristance in memristive cell, enabling cascading without the need for resistance state readout. In comparison to that, VI^3 operations in VI kernel can accomplish the logic function by switching the memristive state directly by applying writing bias on cell terminals in single one cell within single one cycle. The experimental demonstration of VI^3 and MI^3 logic operations by using $BiFeO_3$ memristors are demonstrated in Fig. S4. It includes the memristance of each cell M_{i} before and after logic operations, the voltage applied to the top electrode $V_{\mathrm{TE}}/V_{\mathrm{BLi}}$, the voltage applied to the bottom electrode biases $V_{\mathrm{BE}}/V_{\mathrm{WL}}$, and the absolute value of current from top electrode to bottom electrode through the cell $|I_{\mathrm{Mi}}|/|I_{\mathrm{BLi}}|$.

As depicted in Fig. S4a for VI^3 operation, the resistance state of the cell M_1 is initialized according to m_{x_1} (highlighted in gray) and subsequently verified through a sequential readout operation. This operation involves applying a reading bias of 2 V

across each memristor from top electrode to bottom electrode (highlighted in yellow, consistent across all state checking steps in this study). By employing the voltage combinations v_{x_2} and v_{x_3} to the top electrode and bottom electrode of the memristive cell (indicated in red), the logic output state m_{y_1} is further validated sequentially through readout biases of 2 V applied to the top electrode at the conclusion of the test (highlighted in yellow). As anticipated, the logic output m_{y_1} demonstrates ideal LRS and HRS memristive values according to the '1' or '0' in truth table, attributed to the deterministic switching achieved by applying logic-dependent biases to electrodes on a single cell.

As depicted in Fig. S4b for MI^3 operation, three memristive cells in a WL are sequentially initialized into the resistance state according to $m_{\mathrm{x}_{1-3}}$ (marked in gray), and pre-checked by readout operation sequentially by applying a reading bias of 2 V across each memristor from top electrode to bottom electrode (marked in yellow). By applying $V_{\mathrm{in}} = 5.3$ V on M_2 and M_3 while grounding M_1, the output of MI^3 operation m_{y_1}, which is stored as memristance state in the cell M_1, is further verified sequentially by readout biases of 2 V applied to the top electrode at the end of the test (marked in yellow). It is noteworthy that MI^3 (or VI^3) could also be applied to cells along a BL (rather than WL) by applying reversed biases to bottom electrodes through WL. The switching process in output cell M_1 is observed in logic input combinations '101', '110', and '111', wherein the cell M_1 transitions from its initial LRS to HRS, resulting in a logic output of '0'. For all other logic input combinations, the memristance state remained unchanged before and after the MI^3 operation.

The non-ideal effects of the device significantly influence the performance of the proposed 3-input logic computation method, particularly in MI^3 and VI^3 operations. As discussed in the main article and Supplementary Information A, the greatest impact arises from the switching dynamics and stochastic variability inherent in nanoscale memory devices. Switching dynamics dictate the types of logic gates that can be implemented, with some gates requiring additional processes such as RESET or SET operations, leading to prolonged operation sequences and reduced efficiency. Stochastic variability further exacerbates the issue by introducing high error rates—up to 70% in experimental studies—particularly in cascaded operations. These effects collectively reduce the scalability and accuracy of the computation method. Next, using $\mathrm{BiFeO_3}$-based MI^3 operation as an example (with experimental data shown in Fig. S4), we discuss two major issues in logic designs within the MI kernel, i.e. "Input drift" and "Partial switching" issues. Both issues are inevitable in stateful logic operations. Notably, with much more significant device variability issue in abrupt switching devices compared to analog ones, it further reduces the programming window and leads to an even higher error rate in logic processing.

- "Input drift" issue: Sufficient positive programming bias V_{in} is required for switching output cell to desired memristance state according to truth table. However, too high positive programming bias V_{in} applied to the top electrodes of cells M_2 and M_3 may induce drift in the memristive states towards LRS when the logic input is HRS '0', a phenomenon known as the "input drift" issue. This challenge constitutes one of the primary hurdles in the realization of a stateful logic kernel.

- “Partial switching” issue: Due to the voltage divider effect, with logic input combinations ‘101’, ‘110’, and ‘111’, the insufficient positive bias V_{in} applied to M_2 and M_3 during MI^3 operation, i.e. insufficient positive bias V_{com} to the bottom electrode of M_1 can result in compromised HRS (compromised logic ‘0’) compared to the initial HRS value in BiFeO_3 memristor. This indicates a potential ”partial switching” issue in the output cell inherent to MI^3 logic operations. One approach to mitigathe partial switching issue from ‘1’ to ‘0’ in cell M_1 is to amplify the positive writing bias applied to their top electrodes, thereby elevating the voltage V_{com}. However, this amplification simultaneously increases the resistance value in cell M_1 for the output logic ‘1’ in the logic combination case ‘100’ due to the heightened positive bias applied to its bottom electrode.

Both aforementioned “Input drift” and “Partial switching” issues are inherent in the stateful logic process (cannot be alleviated). Under mixed-mode computing paradigm, our aim is to mitigate these issues through a co-design strategy that enhances computing accuracy, particularly by leveraging the unique self-rectifying and analog switching characteristics of BiFeO_3 memristors. This emphasizes the critical importance of technology-specific optimization in adapting memristor devices for logic designs. Firstly, to ensure stable and accurate outputs, we carefully analyzed the switching kinetics of BiFeO_3 memristors. These kinetics favor easier transitions from LRS to HRS compared to HRS to LRS. Based on this, we structured the MI^3 operation to apply positive bias to the top electrodes of two parallel-connected memristors while grounding the top electrode of the third memristor. This configuration allows us to exploit cascading for “re-initializing” the logic ‘1’, minimizes the influence of partial switching in the input cells, and enhances stability within the cascading MI kernel. Consequently, we designed the MI^3 operation to produce a more reliable ‘0’ output by optimizing the bias voltage V_{in} to 5.3 V. For instance, as demonstrated in experimental results in Fig. S4, the MI^3 operation demonstrates correct transitions from LRS to HRS with input combinations ‘101’, ‘110’, and ‘111’ (HRS of M_1 after MI^3: 111.4 MΩ compared to initialized HRS of M_1 at 402.4 MΩ) as shown in Fig. S4. As expected, with $V_{\text{in}} = 5.3$ V, the MI^3 operation with input combination ‘100’ results in a compromised LRS in M1 after MI^3 (LRS of M_1 after MI^3: 27.2 MΩ compared to initialized LRS of M_1 at 1.1 MΩ). Thus this bias $V_{\text{in}} = 5.3$ V ensures stable ‘0’ outputs across various input combinations, reducing errors in cascaded operations (see experimental results in Fig. S4). As next step, in the definition of M3S automation tool is explicitly designed to allow the output cell of MI^3 operation can only be reassigned as an output cell in subsequent MI^3 operations if it has been used at least once as an input cell in an MI^3 or VI^3 operation. This intentional reassignment avoids scenarios where the output ‘1’ from a prior MI^3 gate is recomputed into another ‘1’ in the next MI^3 cycle, ensures that a more precisely defined input or output value is applied in logic cascading, thereby minimizing error propagation commonly observed in traditional stateful approaches.

D Technology dependency in VI^3 logic operations

As aforementioned, the VO and MI logic kernels are inherently reliant on the underlying technology. However, it has been observed that the 3-input VI^3 operation demonstrates a generic feature: the same logic function can be realized across multiple memristive technologies.

Table S2 Definition of logic input variables in different nanoscale memory devices for realizing 3-input VI^3 logic function.

	m_{x1}: '1'/'0'	v_{x2}:'1'/'0'	v_{x3}: '1'/'0'
Bipolar ReRAM [26]	LRS/HRS	Vw/GND	Vw/GND
Unipolar ReRAM[27]	LRS/HRS	V_{SET}/GND	V_{RESET}/GND
Complementary ReRAM [5]	PLRS(NHRS)/ PHRS(NLRS)	Vw/GND	Vw/GND
PCM [28]	LRS/HRS	Short high voltage Vw/GND	Long medium Vw/GND
STT-MRAM [29]	LRS/HRS	Vw/GND	Vw/GND

This generic feature is because that the design of 3-input VI^3 logic function is rooted from the intrinsic switching behavior of memory devices: the memory device switches from original input state from m_{x_1} to output state m_{y_1} only if the applied voltage/current across the cell (difference between v_{x_2} and v_{x_3}) initiate the underlying switching process.

It is worthy to mention that the performance of the 3-input logic method can vary depending on the underlying device technology. This variation is influenced by factors such as the switching dynamics of the technology, which determine the types of logic gates that can be realized, and device variability, which affects logic accuracy, especially for resistance-controlled logic design. Table S2 demonstrates that the same 3-input VI^3 logic function can be implemented using various series of $BiFeO_3$ memristors, as well as more general types of redox-based random access memory (ReRAM), phase change memory (PCM) [28], and spin transfer torque magneto-resistive RAM (STT-MRAM) [29]. This implementation involves customizing the definitions of v_{x_2} and v_{x_3} to initiate switching based on the specific characteristics of each underlying technology. To adopt the proposed mixed-mode computing architecture, the chosen technology must satisfy two essential criteria: (1) it should enable resistance-controlled logic to support mixed-mode computing, and (2) it should exhibit low device variability to achieve high logic accuracy. Additionally, good endurance is also an important consideration for practical applications. While the selection of the optimal device largely depends on the target application, in general, technologies that offer higher density, lower energy consumption, and faster operation would be most desirable.

E High parallel logic processing in mixed-mode computing

Memristive crossbar configurations offer significant parallel computing capabilities by leveraging the inherent parallelism in their architecture, offering high speed and improved energy efficiency. This section provides a summary of the parallelism computing capabilities inherent in the two-dimensional crossbar architecture, integrated into the design of the M^3S tool.

We exemplify the parallel computing capabilities facilitated by memristive crossbar configurations (Fig. S5), utilizing the proof-of-principle demonstrator N-bit carry-ripple adder with VI^3 and MI^3 as a case study.

For example, as demonstrated in Fig. S6, in the case of the 1-bit carry-ripple adder, each column representing a cell (M_{11}–M_{14}) needed for computation, while M15 stores the output carry bit c1. The five columns represent the five cycles required for completing the 1-bit carry-ripple adder, broken down into three cycles for VI^3 operations and two for MI^3 operations. The VI^3 operations are executed across all cells simultaneously, enabling efficient processing. For each cycle, we specify the WL (Word Line) and BL (Bit Line) inputs used. For implementing 1-bit carry-ripple adder, our mixed-mode approach requires 5 cells and 5 cycles, while a purely in-memory computing approach without reading would require 10 cells and 13 cycles (as referenced in Ref. [22] in Tab.S4 in Supplementary Information I). However, the efficiency gains of the mixed-mode paradigm become more apparent as the bit number increases. For instance, in Fig. S7, the 4-bit carry-ripple adder can be realized in a 4×5 passive crossbar array with 1R configuration, where no readout is allowed in logic cascading. Implementing an 8-bit full adder in our mixed-mode approach requires only 33 cells and 12 cycles, compared to a purely in-memory computing approach, which would require 87 cells and 97 cycles (also as referenced in Ref. [22] in Tab.S4 in Supplementary Information I). This comparison highlights the efficiency of mixed-mode computing in terms of cell and cycle optimization, particularly for multi-bit and complex operations.

Due to the distinct nature of each operation during logic processing, different parallel strategies are possible. Assuming to compute one arbitrary function with logic input variables x_{1_i}, x_{2_i}, and x_{3_i}, with i representing i-th bit in each logic input, the parallel strategies can be described as follows:

- Bitwise parallelism in cascading VI (Fig. S5a): the bit-wise parallelism is facilitated by applying multiple i-th bit of logic inputs to WLs (or BLs) for performing multiple VI operations in a single operational cycle. For example, as demonstrated in Fig. S7, the carry bit c_{i+1} in a modular adder can be iteratively computed based on c_i in cycles 2-5. For computing bit c_1 in BL1, all cells in BL1 are applied by x_{1_1} to all WLs and x_{2_1} to BL1. c_2 can be further computed by applying x_{1_2} to WL2-4 and x_{2_2} to BL1, then c_2 is stored in cells M21, M31 and M41 in BL1. Such bitwise parallelism in cascading VI is particularly suitable in modular computing, where the $(i+1)$-th bit is iteratively computed based on the value of i-th bit.
- All-bit parallelism in cascading VI (Fig. S5b): All-bit parallelism is realized by applying constant input '1' and '0' to BLs (or WLs), and the corresponding WLs

(or BLs) are applied by each bit in logic inputs x_{1_i} in the same cycle. All-bit parallelism is preferred in modular design as it highly reduces the cycle number in n-bit implementations (counted as one cycle). To enable all-bit parallelism, the constant logic input '1'/'0' are preferred as WL or BL operands. As demonstrated in Fig. S7, the all-bit parallelism in cascading VI is explored in cycle 6.

- WL/BL mode parallelism in cascaded MI (Fig. S5c): In the BL mode of cascaded MI operations, parallelism can be achieved by applying logic input independent pulses, such as , $V_{1\text{-}3}$ through three BLs. Typically V_1, $V_2 > V_3$, and the BL cells applied by V_3 store the logic outputs as memristance state after applying MI. The bias differences between V_1,V_2 and V_3 are usually comparable with V_{w}, which represents the amplitude of the writing bias of the cell. All WLs are kept floating during this process. This BL mode operation enables parallel MI operations in three BLs across all floated WLs, including both desired and undesired cells. As demonstrated in Fig. S7, the bitwise parallel operation in the MI kernel in WL mode is also explored in cycle 7 and cycle 8. It is verified that the MI^3 operations demonstrated in the adder design, can apply flexibly any outputs of VI^3 as input cells or output cells, and requires no additional initialization step of the output cells in comparison to conventional MAGIC gate designs.

Note that the parallel computing shown in cascading VI kernel can be utilized also in cascading VO kernel (not shown here).

F Algorithms used in crossbar-oriented mapping and synthesis tool M^{3}S

As has been described in the main Article, M^{3}S takes as inputs the truth tables of Boolean functions $f(x_1, x_2, \ldots)$ to be synthesized and the number of cycles when V-mode and M-mode operations are performed. It then constructs a Boolean satsifiability formula in conjunctive normal form, and solving it using an automatic Boolean satisfiability solver. A conjunctive normal form is comprised of disjunctive clauses over propositional (binary) variables, and its solution entails finding an assignment to the variables that satisfies all clauses [30]. The flowchart of the M^{3}S automation tool is demonstrated in Fig. S8, using an example of the VI kernel and the construction of Eq. 1. As shown in the flowchart, the process begins with the construction of a Boolean formula in conjunctive normal form format, which is input into a Boolean satisfiability solver. The solver determines whether a solution exists and, if successful, the automation tool interprets the solution and implements it on the memristive cells within the crossbar.

Recall that V-mode operations VI^3 have three inputs, one of which is the prior state (resistance) of the same device in the crossbar and two are voltages that come from peripherals. Upon inputting the (single- or multiple-output) Boolean function f into the M^{3}S tool, the constants 0 and 1, and the function f's literals $x_1, \overline{x_1}, \ldots x_n, \overline{x_n}$ (with n being the number of f's inputs), are serving as logic inputs in VI^3 operations, applied to the cell's top electrode and bottom electrode. For simplicity, we assign an index to each available literal function, with constant-0 having index 1. The tool automatically produces a conjunctive normal form defined over variables that represent the minterms

of the realized function f and the outputs of each V-mode and M-mode operations, and a number of additional auxiliary variables. To obtain the optimal solution in terms of the number of required crossbar cells, the designer can start with a small crossbar that has no valid solution and gradually increase its size until the conjunctive normal form is solved. Therefore, the Boolean satisfiability formula contains variables $g^{\mathrm{TE}}_{a,b,j,c}$ and $g^{\mathrm{BE}}_{a,b,j,c}$ for all crossbar locations (a,b) and all cycles c when V-mode operations are executed. $g^{\mathrm{TE}}_{a,b,j,c}$ is set to 1 when the top electrode of the memristor at location (a,b) is driven by literal function l_j; the bottom electrode is analogously described by $g^{\mathrm{BE}}_{a,b,k,c}$. The Boolean satisfiability formula will include, for all M-mode cycles, expressions

$$\bigwedge_{\substack{1\leq j,k\leq 2n+2\\ 1\leq q\leq 2^n}} \left((g^{\mathrm{TE}}_{a,b,j,c} \wedge g^{\mathrm{BE}}_{a,b,k,c}) \rightarrow \left(v_{a,b,c,q} \equiv \mathrm{VI}^3(v_{a,b,c-1,q}, l_{j,q}, l_{k,q})\right)\right). \quad (1)$$

Here, the VI^3 operation in Figure 2c is transformed into conjunctive normal form, as are implication ("$\rightarrow$") and equivalence ("$\equiv$") operators. The variables $g^{\mathrm{TE}}_{a,b,j,c}$ and $g^{\mathrm{BE}}_{a,b,k,c}$ are set to 1 when the top electrode (bottom electrode) of cell M_{ab} is connected to literal function with index j (k). According to the memristance input variable $v_{a,b,c-1,q}$ in M_{ab} in cycle $c-1$, the output variable $v_{a,b,c,q}$ in cycle c is set to 1 when the q-th entry of the VI^3 truth table is 1 and 0 otherwise. For instance, if the solution provides $g^{\mathrm{TE}}_{a,b,j,c} = 1$ while all other $g^{\mathrm{TE}}_{a,b,1,c} = 0$, it signifies that during cycle c, the top electrode of M_{ab} (in BL-b) is driven by $l_1 =$ const-0. Similarly, if $g^{\mathrm{BE}}_{a,b,6,c} = 1$, then the bottom electrode of M_{ab} (in WL-a) is driven by $l_6 = \overline{x_2}$. One more example of Boolean satisfiability formula expressions for this functionality is given in the main Article.

The M-mode operations have three input as well, where each of the input can be either a literal, or the state of any memristor in the crossbar. Let, for simplicity, the list of all allowed input functions be $\alpha_1, \alpha_2, \ldots$, and let the q-th truth table entry of function α_i be $\alpha_{i,q}$. The Boolean satisfiability formula will include, for each crossbar location (a,b) and each M-mode cycle c, three Boolean variables $g^{\mathrm{In1}}_{a,b,j,c}, g^{\mathrm{In2}}_{a,b,j,c}, g^{\mathrm{In3}}_{a,b,k,c}$, where $g^{\mathrm{In}k}_{a,b,j,c}$ is 1 if and only if input k of the memristor at location (a,b) is connected to function α_j during cycle c. It will also include variables $r_{a,b,c,q}$ to represent the q-th entry of the truth table of the function realized on (a,b) during cycle c. Then, the Boolean satisfiability formula will include, for all M-mode cycles, expressions

$$\bigwedge_{a,b,c,q,i,j,k} (g^{\mathrm{In1}}_{a,b,i,c} \wedge g^{\mathrm{In2}}_{a,b,j,c} \wedge g^{\mathrm{In3}}_{a,b,j,c}) \rightarrow \left(r_{a,b,c,q} \equiv \mathrm{MI}^3(\alpha_{i,q}, \alpha_{j,q}, \alpha_{k,q})\right) \quad (2)$$

In addition to expressions describing the V-mode and the M-mode operations, the Boolean satisfiability formula includes clauses to, e.g., enforce that precisely one of the g variables assumes the logical value of 1, i.e., that each operation's inputs are well-defined. In addition, the crossbar structure is enforced by requiring that the inputs of the top electrodes of different memristors on the same column are identical, same with the bottom electrodes of memristors on the same row. (In principle, any particular connectivity beyond crossbars could be expressed as well; e.g., a structure where each memristor is addressable individually would correspond to just removing this last

restriction altogether.) Moreover, the functions at the outputs of the circuit are locked (by means of unit clauses) to the values from the truth table of function f, i.e., the user's specification.

A solution of the constructed Boolean satisfiability formula contains the information which operation input is connected to which literal or output. It also includes all values assumed by the memristor outputs during all considered cycles. If the formula is unsatisfiable, this constitutes a formal proof that no valid circuit is realizable within the given crossbar size and number of V-mode and M-mode cycles. This gives the designer a handle to compute an optimal circuit with a provably minimal size or latency by finding a solution for (possibly too large) crossbar dimensions and number of cycles and then gradually reducing them until the formula becomes unsatisfiable. By incorporating parallel cascading computing in both V- and M-modes within a two-dimensional crossbar setup, M^3S can offer an optimal solution for executing function f while minimizing crossbar size and cycle count. Additionally, it is possible to minimize the number of potentially unreliable M-mode operations, designers can adjust the number of cycles in the second phase, starting from 0, until the satisfiability of the conjunctive normal form produced by M^3S is achieved. Its downside is its limited scalability due to long run times of Boolean satisfiability solving software. This is in line with optimal synthesis methods for classical CMOS technology, which work in practice (except for restricted classes of functions) only for functions with up to 5-6 inputs. Even in the CMOS technology that is capable of realizing circuits with billions of logic gates, optimal synthesis is considered useful because it can be applied hierarchically; we expect the same to hold for the memristive case.

G Proof-of-principle demonstration of N-bit carry-ripple adder exploiting VI^3 and VO^3 Operations

This section showcases the implementation of an N-bit carry-ripple adder utilizing VI^3 and VO^3 operations. Despite the significant power and latency consumption in peripherals associated with readout operations (Supplementary Information B), the VI^3 and VO^3-based N-bit carry-ripple adder solution exhibits remarkably low latency and cell count, surpassing state-of-the-art designs.

Fig. S9 demonstrates the experimental implementation of N-bit carry-ripple adder based on VI^3 and VO^3 by using memristive crossbar based on $BiFeO_3$ cells. The control sequence by M^3S is demonstrated in Fig. S9a. The VI^3 operations are marked with background of red color, including the required readout VO^3 cycle highlighted in dark red, and there is no MI^3 operations needed. One of the eight functions $\{x_1, \bar{x}_1, x_2, \bar{x}_2, x_3, \bar{x}_3$, const-0, const- 1$\}$ is applicable as logic inputs in VI^3 or VO^3 operations at either of the memristive cell's two electrodes with top electrodes controlled by BLs, while bottom electrodes are controlled by WLs. As shown in Fig. S9a, a full carry-ripple adder design by using VI^3 and VO^3 requires 2 rows, indicating 2 cells for implementing 1-bit full adder, including one for computing and storing the output-carry bit c_{i+1}, and the other one for computing and storing output-sum bit

Table S3 Comparison of the implemented N-bit carry-ripple adder based on VI^3 and VO^3 with readout logic and the representative designs by exploiting memory-centric computing with readout logic during cascading.

Logic primitives	# Mem-cells of N-bit adder (N = 8)	# Cycles of N-bit adder (N = 8)	Area-Delay-Product with N = 8	Synthesis methodology	Full adder types	References, year
RIMP, NIMP, XOR (2-input logic)	2(N+1) 18	2N+4 20	360	Handcraft	Carry ripple adder	[31], 2015
XOR, NAND (2-input logic)	2N+1 17	2N+1 17	289	Handcraft	Carry ripple adder	[32], 2018
RIMP/NIMP (2-input logic)	$(5 + \log_2(N)) \cdot N$ 64	$(21 + \log_2(N) - 1) \cdot 8$ 37	2368	Handcraft	Sklansky Tree adder	[33], 2019
XOR+Majority (2-input logic)	3N 24	2N+2 18	432	Standard tool	Carry ripple adder	[34], 2021
Majority, NOT (2-input logic)	$(8N + 16) \cdot 6$* 480*	$4\log_2(N) + 6$ 18	8640	Standard tool	Parallel-prefix adder	[35], 2021
XOR+Majority (2-input logic)	10N+1 81	8+2N 24	1944	Handcraft	Carry ripple adder	[36], 2023
VI^3,VO^3 (3-input logic)	N+1 9	N+4 12	108	M^3S tool	Carry ripple adder	This work

* The area cost of sense amplifier has been considered and included by the authors.

s_i. The 5 numbered columns indicate the 5 cycles required for implementing 1-bit full adder, 4 of VI^3 operations (marked in light red) and 1 of VO^3 (marked in dark red).

Starting with unknown state x, the c_{i+1}, computed through VI^3 operations during cycles 1 and 2, are stored as memristive states within both cells. Subsequently, c_{i+1} is retrieved via a non-destructive VO^3 operation, with logic inputs $v_{x2} = \bar{v}_{x3} = 1$, during cycle 3. This readout value, c_{i+1}, further serves as v_{x3} in the subsequent VI^3 operation during cycle 5 for the computation of s_i on cell 2, on which the value s'_i is computed and stored by VI^3 during cycle 4.

As an example, the 4-bit carry-ripple adder with input $a = x_2 = 0011$ and $b = x_3 = 0101$ is experimentally tested on a passive crossbar based on $BiFeO_3$ memristive cells. In order to maximize the parallelism, we implement the automation flow in Fig. S9a in the diagonal cells of a crossbar array. Starting with c_0, all carry bits c_{i+1} are computed by bitwise parallel VI^3 operations (cycles 2-5), and readout in cycle 6 in parallel from WLs. The sum bits of s'_i (cycle 7) and s_i (cycle 8) are computed by the parallel VI^3 operations on all bits. Fig. S9c shows the memristance, voltages on WLs and BLs, and absolute values of current across the required 5 cells during 8 cycles. The carry bit c_4 is computed and stored in cell M55. The correctness of output states is verified by applying reading biases as 2 V to top electrode and 0 V to bottom electrode in each output cell; the observed states $s_{0\text{-}3}$ and c_4 are indicated in the diagram.

N-bit carry-ripple adder implementation based on VI^3 and VO^3 can be considered as an area-optimized solution, which requires the minimum possible $N + 1$ cells for processing a N-bit adder. Only the iterative computation of c_{i+1} has to be repeated for each input bit of a and b. The cell number required by N-bit carry-ripple adder based on VI^3 and VO^3 is limited by the number of computing bits N in addition processing. The readout of c_{i+1} in cycle 3, the computation of s'_i (cycle 4) and s_i (cycle 5) can be

realized in a parallel manner (counted as 1 cycle for N bit). Computing N-bit carry-ripple adder requires $N+1$ cells (one for saving the output-carry bit $c_{\mathrm{i+1}}$) and $N+4$ cycles, while maximizing the parallelism during VI^3 VO^3 operations.

However, it is noteworthy that to leverage diagonal cells for N-bit carry-ripple adder will block all the cells in the applied WLs and BLs, even there is no operation required on them. As an alternative, the N-bit adder in Fig. S9a can also be implemented by applying memristive cells in one WL or BL, reaching the minimum possible $N+1$ cells with slightly increased cycle number of $3N+1$ cycles (not shown here). The comparison of the proposed N-bit carry-ripple adder based on VI^3 and VO^3 with the previously published adders is summarized in Tab. S3. The proposed N-bit carry-ripple adder based on VI^3 and VO^3 demonstrates the best computing performance in terms of cell number and cycle number in comparison to state-of-the-art approaches.

H Energy evaluation in mixed-mode computing

A primary advantage of memristive devices over CMOS is their ability to combine both logic and memory functions within a single device, which is particularly relevant for logic-in-memory computing schemes. As data processing requirements grow, the inefficiencies of moving data back and forth between memory and the CPU in traditional von Neumann architectures become increasingly problematic. Data movement between the memory and processor in CMOS-based systems can consume up to 1000 times more energy than the logic operations themselves. In fact, this energy limitation is one of the most pressing issues in the computing industry as the energy required to compute grows exponentially while the global energy production grows only linearly, year after year. As noted in Supplementary Information D, our approach is transferable to a variety of memristive devices, and the performance of our approach is closely tied to the properties of these devices. Substantial advancements in memristor technology are actively being made. Recent progress includes write speeds reaching 20 ps [37], feature sizes down to 2x2 nm^2 [38], multilevel switching capabilities with over 2048 discrete levels in a single device [39], and stackability of up to 8 layers for 3D integration [40]. These advancements hint at future improvements in speed, energy efficiency, and scalability for memristor-based computing. In literature it was shown that hybrid computing concepts offloading some of the processing in the memory, in logic in memory applications [41, 42] and in neuromorphic computing applications [43–47], leads to performance improvements in particular by reducing the amount of data to be moved between memory and CPU. Building on these advancements, we further emphasize that our approach integrates both memristance (M) and voltage (V) as active logic variables within a single computational process. This mixed-mode operation introduces computing capabilities that were previously unexplored. In contrast to existing in-memory computing methods, which utilize either memristance or voltage for logic computation (e.g., Refs. [41–47]), our approach incorporates both, providing an additional degree of freedom to computing architectures. Rather than replacing conventional in-memory logic, our method augments existing approaches, potentially unlocking additional computational possibilities while carefully managing switching overhead. For instance, our approach can be particularly

beneficial for hyperdimensional computing (HDC) [48], which has shown great promise in machine learning tasks such as text classification, biomedical signal processing, and sensor fusion. HDC operations like binding and bundling can be efficiently performed in-memory, with writing-based operations limited to preparing the associative memory array, thereby reducing endurance concerns. As reported in Ref. [48], memristors are utilized for in-memory logic operations for binding. Unlike prior approaches that rely on memristor-based XOR lookup tables or digital logic gates, our mixed-mode approach leverages both voltage and memristance as logic variables, enhancing computational efficiency. To make memory technology competitive with conventional technologies, several critical factors must be addressed: 1) Endurance Considerations: The endurance of memristors is a critical factor in memory technology. While conventional non-volatile memory technologies require high endurance ($\geq 10^{16}$ cycles) for practical development, our approach can mitigate endurance concerns through distributed and parallel switching strategies. For instance, if we assume sequential additions in a memory block, individual devices would only switch a limited number of times. The endurance requirement can be approximated as:

$$Required\ endurance = \frac{\#switching\ cycles\ per\ device}{\#operations\ performed} \tag{3}$$

By efficiently distributing operations across memory cells and utilizing techniques such as write-sharing, the required endurance per device can be significantly reduced, making the approach more practical. 2) Energy Efficiency Considerations: A key metric for competitiveness is that the energy cost of performing operations must be lower than the energy required for moving data to the CPU. We estimate energy consumption based on the number of switching events:

$$E_{op} \leq \frac{E_{data\ movement}}{\#switching\ events} \tag{4}$$

Additionally, charging and discharging the crossbar array introduce additional energy overheads, as described in prior studies (e.g., [49]). In such cases, optimizing the frequency of charge/discharge cycles is crucial to maintaining energy efficiency. Future designs incorporating smarter charge management strategies and optimizing peripheral circuitry will further enhance efficiency.

In the context of the mixed-mode computing paradigm, the energy cost associated with logic computation is heavily influenced by the characteristics of the underlying memristive technology, particularly its switching kinetics. For a specific device technology, this energy cost is further dictated by the number of write and readout operations required for a given implementation. As memristive technology continues to advance, these developments will likely enable even greater efficiency and scalability for mixed-mode and logic-in-memory computing paradigms. Given the adaptability of mixed-mode computing, facilitated by crossbar-oriented automatic flow tools that seamlessly accommodate diverse technologies and facilitate transferability, we assess the energy consumption of logic computation within this paradigm by quantifying the number of write and readout operations. The N-bit carry-ripple adder based on VI^3

and MI^3 demonstrated in Fig. 3 requires $\left(3N^2 + 21N + 2\right)/2$ programming operations (write operations) and no readout operations during logic cascading, whereas the N-bit carry-ripple adder based on VI^3 and VO^3 in Fig. S9 requires in total $\left(N^2 + 7N + 2\right)/2$ programming operations as well as N read operations. For example, if the energy cost of readout VO kernel is the same as programming exploiting MI kernel, the adder with readout will always be more efficient. However, as discussed in Supplementary Information B, in practical cases, the readout VO kernel requires additional power and latency from peripherals, e.g. the output dependent control logics, makes it more expensive than programming operations in MI kernel. Thus, while assuming a constant average energy cost of each programming operation, the overall energy cost of N-bit carry-ripple adder is not only depending on the ratio of energy required for reading and programming on N in the memory cells, but also strongly influenced by the complexity in the individual peripheral design surrounding the memory blocks.

I Comparison with state-of-the-art implementation of full adders

Table S4 presents a comprehensive comparison of representative N-bit full adder designs alongside adder design proposed in this study. Notably, all existing and representative works featured in the comparison table leverage memristive logic designs from either the MI or VI kernel. In contrast, our work distinguishes itself as the first to exploit both kernels for arithmetic logic processing. As promised, our approach, employing mixed-mode logic processing through VI^3 and MI^3 operations, showcases an optimal balance between cell and cycle numbers, evident from the Area-Delay-Product column for 8-bit adders.

It is crucial to emphasize that, unlike the other entries in the list which are simulation-based studies on addition processing without readout, our work stands out as experimental, as indicated by the bold entry in the final row. Comparing Ref. [22] with our approach, it is true that the area-delay product of our approach is more advantageous for small- to medium-bit adders but becomes less favorable beyond 14-bit implementations. However, there are important trade-offs that must be considered: the approach in Ref. [22] does not inherently store the result of the computation, which means that additional storage would be required if result storage is necessary, leading to an area overhead that also scales with N. Furthermore, it is noteworthy that only few studies demonstrating experimental implementations of addition functions in the existing literature, due to the challenges posed by reliability issues in memristor cells (Supplementary Information C). For instance, Ref. [60] presents the experimental implementation of individual logic gate designs, with the full adder demonstrated via simulation by leveraging these experimental results. Ref. [61] presents an experimental study on an 8-bit full adder employing a three-dimensional stack of monolithically integrated bipolar memristors. Such robust and optimized IMPLY logic designs is achieved by substituting the load resistor with a current source, though at the expense of compatibility with the conventional crossbar structure.

Table S4 Comparison of the implementation for N-bit adders between the mixed-mode approaches of this work and the representative ones by exploiting memory-centric computing (no readout during logic cascading).

Logic operations (x-input logic[1])	Logic kernel classification	# Mem-cells of N-bit adder (N = 8)	# cycles of N-bit adder (N = 8)	# Other devices of N-bit adder (N = 8)	Area-Delay-Product (8-bit adder)	Full adder types	Synthesis methodology	Array compatible?	References, published year
IMPLY,FALSE (2-input logic)	MI	3N+5 (29)	88N+48 (252)	Resistor: 1 (1)	22560	Carry ripple adder	Standard tool	Y	[50], 2009
IMPLY,FALSE (2-input logic)	MI	3N+3 (27)	29N (232)	Resistor: 1 (1)	6496	Carry ripple adder	Standard tool, optimized for series operation	Y	[23], 2013
IMPLY, FALSE (2-input logic)	MI	9N (72)	5N+18 (58)	Resistor: 1 (1)	4234	Carry ripple adder	Standard tool, optimized for parallel operation	Y*	[23], 2013
AND, OR, NOT (2-input logic)	VI	- (128)	- (1)	Transistors: - (112)	240	Carry ripple adder	Standard tool with optimized algorithm for cell number	N	[51], 2015
NOR (2-input logic)	MI	5	15N (120)	-	600	Carry ripple adder	Standard tool with area optimized	Y	[22], 2016
NOR (2-input logic)	MI	11N-1 (87)	12N+1 (97)	-	8439	Carry ripple adder	Standard tool with latency optimized	Y	[22], 2016
IMPLY, FALSE (2-input logic)	MI	2N+3 (19)	22N (176)	Resistor: 1 (1)	3520	Carry ripple adder	optimized algorithm specific for CRA	Y	[24], 2017
NOR, NOT (2-input logic)	MI	- (N = 4: 48)	- (N = 4: 101)	-	4848 (N = 4)	Carry look-ahead adder	Standard tool	Y	[52], 2017
NOR, NOT (2-input logic)	MI	14N+1 (113)	12N+1 (97)	-	2793	Carry ripple adder	Standard tool	Y	[52], 2017
AND, OR, NOT (2-input logic)	VI	- (228)	1 (1)	Transistors: - (64)	292	Carry save adder	Standard tool with area optimized	N	[53], 2019
IMPLY, OR, NOR, COPY operation (2-input logic)	MI	11N (88)	6N+6 (54)	Resistor: 1 (1)	4806	Carry ripple adder	Handcraft	Y	[25], 2019
IMPLY, FALSE (2-input logic)	MI	2N+3 (19)	17N (136)	Resistor: 1 (1)	2720	Carry ripple adder	Standard tool optimized for semiparallel processing	Y*	[54], 2019
IMPLY, FALSE COPY operation (3-input logic)	MI	6N+6 (54)	2N+15 (31)	Resistor: 1 (1)	1705	Carry ripple adder	Handcraft	Y	[55], 2019
IMPLY, AND (2-input logic)	MI	5N+1 (41)	6N+8 (56)	Resistor: 1 (1)	2352	Carry ripple adder	Handcraft	Y*	[56], 2020
AND, OR, NOT (2-input logic)	VI	18N (144)	1 (1)	Transistors: 8N (64)	208	Carry ripple adder	Standard tool, optimized for energy and step delay	Y*	[57], 2021
XOR (2-input logic**)	MI	6N+3 (51)	2N+2 (18)	-	918	Carry ripple adder	Standard tool	Y*	[9], 2021
IMPLY, FALSE (2-input logic)	MI	5N+1 (41)	N+4 (12)	MOSFETs: 8N+1 (65)	1272	Carry ripple adder	Handcraft	Y*	[58], 2022
IMPLY, COPY operation (2-input logic)	MI	19(N/2)+6 (82)	3N+27 (51)	Resistor: 1 (1)	4233	Carry select adder	Standard tool	Y	[59], 2023
IMPLY, COPY operation (2-input logic)	MI	- (136)	- (54)	Resistor: 1 (1)	7398	Conditional carry adder	Standard tool	Y	[59], 2023
**MI^3, VI^3 operations (3-input logic)**	**MI, VI**	**4N+1 (33)**	**N+4 (12)**	**-**	**396**	**Carry ripple adder**	**M^3S tool**	**Y**	**This work**

[1]x determines the number of logic input variables in the gate design.

* The works in [23], [54], [57] and [9] require significant structural modification. The work in [56] require 3D array with antiparallel memristors. The work in [58] require combined arrays.

It is further noteworthy that all full adder designs using the VI kernel [51, 53, 57] exhibit superior Area-Delay-Product for 8-bit adders, due to the single cycle processing. However, they all necessitate significant structural modifications or require CMOS gates for practical implementation, thus lack crossbar compatibility. Moreover, most studies exploiting MI kernel (including the Refs. [22–24, 50, 52, 54, 56, 58] listed in the Table S4) have often assumed precise alignment of inputs with designed logic gates. However, data distribution within a memristor crossbar can span multiple locations, necessitating in-memory data relocation such as copying or transmission within the crossbar, leading to inevitable additional power costs. In contrast, we employ the VO kernel to transmit the existing logic input variables stored in the crossbar to peripherals before cascading operations commence. This approach eliminates the need for data copying and transmission, and further enables the VI kernel.

References

[1] Borghetti, J., Snider, G.S., Kuekes, P.J., Yang, J.J., Stewart, D.R., Williams, R.S.: 'memristive'switches enable 'stateful'logic operations via material implication. Nature **464**(7290), 873–876 (2010)

[2] Kvatinsky, S., Belousov, D., Liman, S., Satat, G., Wald, N., Friedman, E.G., Kolodny, A., Weiser, U.C.: Magic—memristor-aided logic. IEEE Transactions on Circuits and Systems II: Express Briefs **61**(11), 895–899 (2014)

[3] Papandroulidakis, G., Vourkas, I., Vasileiadis, N., Sirakoulis, G.C.: Boolean logic operations and computing circuits based on memristors. IEEE Transactions on Circuits and Systems II: Express Briefs **61**(12), 972–976 (2014)

[4] Linn, E., Rosezin, R., Kügeler, C., Waser, R.: Complementary resistive switches for passive nanocrossbar memories. Nature materials **9**(5), 403–406 (2010)

[5] You, T., Shuai, Y., Luo, W., Du, N., Bürger, D., Skorupa, I., Hübner, R., Henker, S., Mayr, C., Schüffny, R., *et al.*: Exploiting memristive bifeo3 bilayer structures for compact sequential logics. Advanced Functional Materials **24**(22), 3357–3365 (2014)

[6] Kvatinsky, S., Wald, N., Satat, G., Kolodny, A., Weiser, U.C., Friedman, E.G.: Mrl—memristor ratioed logic. In: 2012 13th International Workshop on Cellular Nanoscale Networks and Their Applications, pp. 1–6 (2012). IEEE

[7] Vourkas, I., Sirakoulis, G.C.: A novel design and modeling paradigm for memristor-based crossbar circuits. IEEE Transactions on Nanotechnology **11**(6), 1151–1159 (2012)

[8] Xie, L., Du Nguyen, H.A., Yu, J., Kaichouhi, A., Taouil, M., AlFailakawi, M., Hamdioui, S.: Scouting logic: A novel memristor-based logic design for resistive computing. In: 2017 IEEE Computer Society Annual Symposium on VLSI (ISVLSI), pp. 176–181 (2017). IEEE

[9] TaheriNejad, N.: Sixor: Single-cycle in-memristor xor. IEEE Transactions on Very Large Scale Integration (VLSI) Systems **29**(5), 925–935 (2021)

[10] Gupta, S., Imani, M., Rosing, T.: Felix: Fast and energy-efficient logic in memory. In: 2018 IEEE/ACM International Conference on Computer-Aided Design (ICCAD), pp. 1–7 (2018). IEEE

[11] Kim, Y.S., Son, M.W., Kim, K.M.: Memristive stateful logic for edge boolean computers. Advanced Intelligent Systems **3**(7), 2000278 (2021)

[12] Hoffer, B., Rana, V., Menzel, S., Waser, R., Kvatinsky, S.: Experimental demonstration of memristor-aided logic (magic) using valence change memory (vcm). IEEE Transactions on Electron Devices **67**(8), 3115–3122 (2020)

[13] In, J.H., Kim, Y.S., Song, H., Kim, G.M., An, J., Jeon, J.B., Kim, K.M.: A universal error correction method for memristive stateful logic devices for practical near-memory computing. Advanced Intelligent Systems **2**(9), 2000081 (2020)

[14] Kim, Y.S., Son, M.W., Song, H., Park, J., An, J., Jeon, J.B., Kim, G.Y., Son, S., Kim, K.M.: Stateful in-memory logic system and its practical implementation in a taox-based bipolar-type memristive crossbar array. Advanced Intelligent Systems **2**(3), 1900156 (2020)

[15] Du, N., Manjunath, N., Li, Y., Menzel, S., Linn, E., Waser, R., You, T., Bürger, D., Skorupa, I., Walczyk, D., *et al.*: Field-driven hopping transport of oxygen vacancies in memristive oxide switches with interface-mediated resistive switching. Physical review applied **10**(5), 054025 (2018)

[16] Shuai, Y., Zhou, S., Bürger, D., Helm, M., Schmidt, H.: Nonvolatile bipolar resistive switching in au/bifeo3/pt. Journal of Applied Physics **109**(12), 124117 (2011) https://doi.org/10.1063/1.3601113 https://pubs.aip.org/aip/jap/article-pdf/doi/10.1063/1.3601113/13287575/124117_1_online.pdf

[17] Shuai, Y., Du, N., Ou, X., Luo, W., Zhou, S., Schmidt, O.G., Schmidt, H.: Improved retention of nonvolatile bipolar bifeo3 resistive memories validated by memristance measurements. physica status solidi c **10**(4), 636–639 (2013) https://doi.org/10.1002/pssc.201200881 https://onlinelibrary.wiley.com/doi/pdf/10.1002/pssc.201200881

[18] You, T., Shuai, Y., Luo, W., Du, N., Bürger, D., Skorupa, I., Hübner, R., Henker, S., Mayr, C., Schüffny, R., Mikolajick, T., Schmidt, O.G., Schmidt, H.: Exploiting memristive bifeo3 bilayer structures for compact sequential logics. Advanced Functional Materials **24**(22), 3357–3365 (2014) https://doi.org/10.1002/adfm.201303365 https://onlinelibrary.wiley.com/doi/pdf/10.1002/adfm.201303365

[19] Du, N., Kiani, M., Mayr, C.G., You, T., Bürger, D., Skorupa, I., Schmidt, O.G., Schmidt, H.: Single pairing spike-timing dependent plasticity in bifeo3 memristors

with a time window of 25 ms to 125 μs. Frontiers in Neuroscience **9** (2015) https://doi.org/10.3389/fnins.2015.00227

[20] Zhao, X., Ruchti, J., Frisch, C., Li, K., Chen, Z., Menzel, S., Waser, R., Schmidt, H., Polian, I., Pehl, M., et al.: Understanding stochastic behavior of self-rectifying memristors for error-corrected physical unclonable functions. IEEE Transactions on Nanotechnology (2024)

[21] Bengel, C., Cüppers, F., Payvand, M., Dittmann, R., Waser, R., Hoffmann-Eifert, S., Menzel, S.: Utilizing the switching stochasticity of hfo2/tiox-based reram devices and the concept of multiple device synapses for the classification of overlapping and noisy patterns. Frontiers in neuroscience **15**, 661856 (2021)

[22] Talati, N., Gupta, S., Mane, P., Kvatinsky, S.: Logic design within memristive memories using memristor-aided logic (magic). IEEE Transactions on Nanotechnology **15**(4), 635–650 (2016)

[23] Kvatinsky, S., Satat, G., Wald, N., Friedman, E.G., Kolodny, A., Weiser, U.C.: Memristor-based material implication (imply) logic: Design principles and methodologies. IEEE Transactions on Very Large Scale Integration (VLSI) Systems **22**(10), 2054–2066 (2013)

[24] Rohani, S.G., TaheriNejad, N.: An improved algorithm for imply logic based memristive full-adder. In: 2017 IEEE 30th Canadian Conference on Electrical and Computer Engineering (CCECE), pp. 1–4 (2017). IEEE

[25] Cheng, L., Li, Y., Yin, K.-S., Hu, S.-Y., Su, Y.-T., Jin, M.-M., Wang, Z.-R., Chang, T.-C., Miao, X.-S.: Functional demonstration of a memristive arithmetic logic unit (memalu) for in-memory computing. Advanced Functional Materials **29**(49), 1905660 (2019)

[26] Jin, L., Shuai, Y., Ou, X., Luo, W., Wu, C., Zhang, W., Bürger, D., Skorupa, I., You, T., Du, N., *et al.*: Transport properties of ar+ irradiated resistive switching bifeo3 thin films. Applied Surface Science **336**, 354–358 (2015)

[27] Yin, L., Cheng, R., Wang, Z., Wang, F., Sendeku, M.G., Wen, Y., Zhan, X., He, J.: Two-dimensional unipolar memristors with logic and memory functions. Nano letters **20**(6), 4144–4152 (2020)

[28] Burr, G.W., Breitwisch, M.J., Franceschini, M., Garetto, D., Gopalakrishnan, K., Jackson, B., Kurdi, B., Lam, C., Lastras, L.A., Padilla, A., *et al.*: Phase change memory technology. Journal of Vacuum Science & Technology B **28**(2), 223–262 (2010)

[29] Kültürsay, E., Kandemir, M., Sivasubramaniam, A., Mutlu, O.: Evaluating stt-ram as an energy-efficient main memory alternative. In: 2013 IEEE International Symposium on Performance Analysis of Systems and Software (ISPASS), pp.

256–267 (2013). IEEE

[30] Biere, A., Heule, M., Maaren, H., Walsh, T. (eds.): Handbook of Satisfiability - Second Edition. Frontiers in Artificial Intelligence and Applications, vol. 336. IOS Press, Amsterdam, Netherlands (2021). https://doi.org/10.3233/FAIA336 . https://doi.org/10.3233/FAIA336

[31] Siemon, A., Menzel, S., Waser, R., Linn, E.: A complementary resistive switch-based crossbar array adder. IEEE journal on emerging and selected topics in circuits and systems **5**(1), 64–74 (2015)

[32] Wang, Z.-R., Li, Y., Su, Y.-T., Zhou, Y.-X., Cheng, L., Chang, T.-C., Xue, K.-H., Sze, S.M., Miao, X.-S.: Efficient implementation of boolean and full-adder functions with 1t1r rrams for beyond von neumann in-memory computing. IEEE Transactions on Electron Devices **65**(10), 4659–4666 (2018)

[33] Siemon, A., Menzel, S., Bhattacharjee, D., Waser, R., Chattopadhyay, A., Linn, E.: Sklansky tree adder realization in 1s1r resistive switching memory architecture. The European Physical Journal Special Topics **228**, 2269–2285 (2019)

[34] Pinto, F., Vourkas, I.: Robust circuit and system design for general-purpose computational resistive memories. Electronics **10**(9), 1074 (2021)

[35] Reuben, J., Pechmann, S.: Accelerated addition in resistive ram array using parallel-friendly majority gates. IEEE Transactions on Very Large Scale Integration (VLSI) Systems **29**(6), 1108–1121 (2021)

[36] Brackmann, L., Ziegler, T., Jafari, A., Wouters, D.J., Tahoori, M.B., Menzel, S.: Improved arithmetic performance by combining stateful and non-stateful logic in resistive random access memory 1t–1r crossbars. Advanced Intelligent Systems **6**(3), 2300579 (2024)

[37] Csontos, M., Horst, Y., Olalla, N.J., Koch, U., Shorubalko, I., Halbritter, A., Leuthold, J.: Picosecond time-scale resistive switching monitored in real-time. Advanced Electronic Materials **9**(6), 2201104 (2023) https://doi.org/10.1002/aelm.202201104 https://onlinelibrary.wiley.com/doi/pdf/10.1002/aelm.202201104

[38] Pi, S., Li, C., Jiang, H., Xia, W., Xin, H., Yang, J.J., Xia, Q.: Memristor crossbar arrays with 6-nm half-pitch and 2-nm critical dimension. Nature Nanotechnology **14**(1), 35–39 (2019) https://doi.org/10.1038/s41565-018-0302-0

[39] Rao, M., Tang, H., Wu, J., Song, W., Zhang, M., Yin, W., Zhuo, Y., Kiani, F., Chen, B., Jiang, X., Liu, H., Chen, H.-Y., Midya, R., Ye, F., Jiang, H., Wang, Z., Wu, M., Hu, M., Wang, H., Xia, Q., Ge, N., Li, J., Yang, J.J.: Thousands of conductance levels in memristors integrated on cmos. Nature **615**(7954), 823–829

(2023) https://doi.org/10.1038/s41586-023-05759-5

[40] Lin, P., Li, C., Wang, Z., Li, Y., Jiang, H., Song, W., Rao, M., Zhuo, Y., Upadhyay, N.K., Barnell, M., Wu, Q., Yang, J.J., Xia, Q.: Three-dimensional memristor circuits as complex neural networks. Nature Electronics **3**(4), 225–232 (2020) https://doi.org/10.1038/s41928-020-0397-9

[41] Le Gallo, M., Sebastian, A., Mathis, R., Manica, M., Giefers, H., Tuma, T., Bekas, C., Curioni, A., Eleftheriou, E.: Mixed-precision in-memory computing. Nature Electronics **1**(4), 246–253 (2018) https://doi.org/10.1038/s41928-018-0054-8

[42] Le Gallo, M., Sebastian, A., Cherubini, G., Giefers, H., Eleftheriou, E.: Compressed sensing with approximate message passing using in-memory computing. IEEE Transactions on Electron Devices **65**(10), 4304–4312 (2018) https://doi.org/10.1109/TED.2018.2865352

[43] Le Gallo, M., Hrynkevych, O., Kersting, B., Karunaratne, G., Vasilopoulos, A., Khaddam-Aljameh, R., Syed, G.S., Sebastian, A.: Demonstration of 4-quadrant analog in-memory matrix multiplication in a single modulation. npj Unconventional Computing **1**(1), 11 (2024) https://doi.org/10.1038/s44335-024-00010-4

[44] Ankit, A., Hajj, I.E., Chalamalasetti, S.R., Ndu, G., Foltin, M., Williams, R.S., Faraboschi, P., Hwu, W.-m.W., Strachan, J.P., Roy, K., Milojicic, D.S.: Puma: A programmable ultra-efficient memristor-based accelerator for machine learning inference. In: Proceedings of the Twenty-Fourth International Conference on Architectural Support for Programming Languages and Operating Systems. ASPLOS '19, pp. 715–731. Association for Computing Machinery, New York, NY, USA (2019). https://doi.org/10.1145/3297858.3304049 . https://doi.org/10.1145/3297858.3304049

[45] Wan, W., Kubendran, R., Schaefer, C., Eryilmaz, S.B., Zhang, W., Wu, D., Deiss, S., Raina, P., Qian, H., Gao, B., Joshi, S., Wu, H., Wong, H.-S.P., Cauwenberghs, G.: A compute-in-memory chip based on resistive random-access memory. Nature **608**(7923), 504–512 (2022) https://doi.org/10.1038/s41586-022-04992-8

[46] Huang, Y., Ando, T., Sebastian, A., Chang, M.-F., Yang, J.J., Xia, Q.: Memristor-based hardware accelerators for artificial intelligence. Nature Reviews Electrical Engineering **1**(5), 286–299 (2024) https://doi.org/10.1038/s44287-024-00037-6

[47] Shafiee, A., Nag, A., Muralimanohar, N., Balasubramonian, R., Strachan, J.P., Hu, M., Williams, R.S., Srikumar, V.: Isaac: A convolutional neural network accelerator with in-situ analog arithmetic in crossbars. In: 2016 ACM/IEEE 43rd Annual International Symposium on Computer Architecture (ISCA), pp. 14–26 (2016). https://doi.org/10.1109/ISCA.2016.12

[48] Karunaratne, G., Gallo, M.L., Cherubini, G., Benini, L., Rahimi, A., Sebastian, A.: In-memory hyperdimensional computing. Nature Electronics **3**(6), 327–337

(2020) https://doi.org/10.1038/s41928-020-0410-3

[49] Zhirnov, V.V., Cavin, R.K., Menzel, S., Linn, E., Schmelzer, S., Bräuhaus, D., Schindler, C., Waser, R.: Memory devices: Energy–space–time tradeoffs. Proceedings of the IEEE **98**(12), 2185–2200 (2010) https://doi.org/10.1109/JPROC.2010.2064271

[50] Lehtonen, E., Laiho, M.: Stateful implication logic with memristors. In: 2009 IEEE/ACM International Symposium on Nanoscale Architectures, pp. 33–36 (2009). IEEE

[51] Liu, B., Wang, Y., You, Z., Han, Y., Li, X.: A signal degradation reduction method for memristor ratioed logic (mrl) gates. IEICE Electronics Express **12**(8), 20150062–20150062 (2015)

[52] Thangkhiew, P., Gharpinde, R., Yadav, D.N., Datta, K., Sengupta, I.: Efficient implementation of adder circuits in memristive crossbar array. In: TENCON 2017-2017 IEEE Region 10 Conference, pp. 207–212 (2017). IEEE

[53] Mandal, S., Sinha, J., Chakraborty, A.: Design of memristor–cmos based logic gates and logic circuits. In: 2019 2nd International Conference on Innovations in Electronics, Signal Processing and Communication (IESc), pp. 215–220 (2019). IEEE

[54] Rohani, S.G., Taherinejad, N., Radakovits, D.: A semiparallel full-adder in imply logic. IEEE Transactions on Very Large Scale Integration (VLSI) Systems **28**(1), 297–301 (2019)

[55] Siemon, A., Drabinski, R., Schultis, M., Hu, X., Linn, E., Heittmann, A., Waser, R., Querlioz, D., Menzel, S., Friedman, J.: Stateful three-input logic with memristive switches. Scientific reports **9**(1), 14618 (2019)

[56] Xu, N., Park, T.G., Kim, H.J., Shao, X., Yoon, K.J., Park, T.H., Fang, L., Kim, K.M., Hwang, C.S.: A stateful logic family based on a new logic primitive circuit composed of two antiparallel bipolar memristors. Advanced Intelligent Systems **2**(1), 1900082 (2020)

[57] Ali, K.A., Rizk, M., Baghdadi, A., Diguet, J.-P., Jomaah, J.: Hybrid memristor–cmos implementation of combinational logic based on x-mrl. Electronics **10**(9), 1018 (2021)

[58] Fu, X., Li, Q., Wang, W., Xu, H., Wang, Y., Wang, W., Yu, H., Li, Z.: High-speed memristor-based ripple carry adders in 1t1r array structure. IEEE Transactions on Circuits and Systems II: Express Briefs **69**(9), 3889–3893 (2022)

[59] Kaushik, N., Srinivasu, B.: Imply-based high-speed conditional carry and carry select adders for in-memory computing. IEEE Transactions on Nanotechnology

(2023)

[60] Kim, K.M., Williams, R.S.: A family of stateful memristor gates for complete cascading logic. IEEE Transactions on Circuits and Systems I: Regular Papers **66**(11), 4348–4355 (2019)

[61] Adam, G.C., Hoskins, B.D., Prezioso, M., Strukov, D.B.: Optimized stateful material implication logic for three-dimensional data manipulation. Nano Research **9**, 3914–3923 (2016)

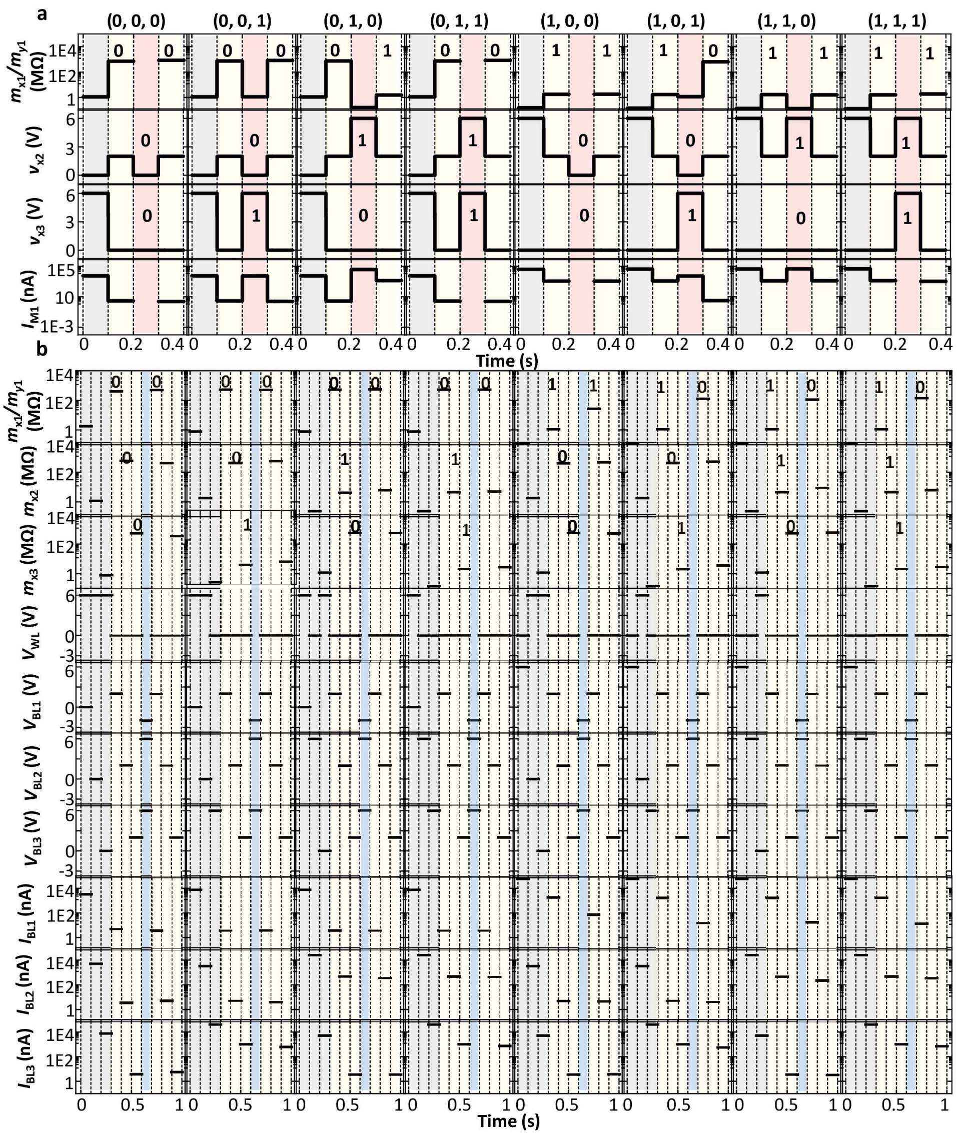

Fig. S4 Extended experimental demonstration of (a) VI^3 and (b) MI^3 logic operations by using $BiFeO_3$ memristive crossbar.

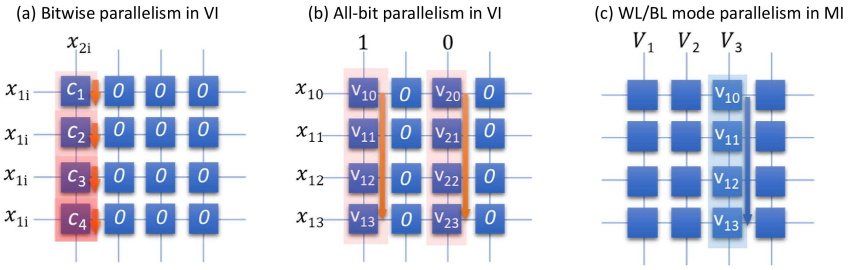

Fig. S5 Illustration of parallelism computing facilitated by memristive crossbar configurations.

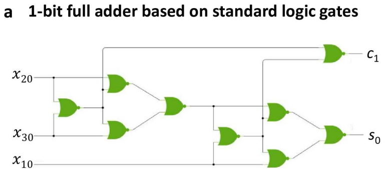

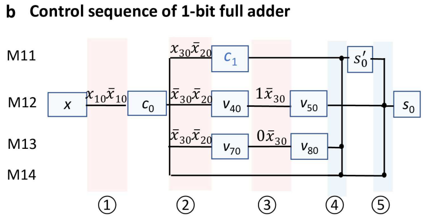

c a = x_{20} , b = x_{30} , c = x_{10}

Cycle	M11	M12	M13	M14	M15
BL_i (TE)	x_{10}	x_{10}	x_{10}	x_{10}	x_{10}
1	x	x	x	x	x
WL_1 (BE)	$\bar{x}_{10}$	$\bar{x}_{10}$	$\bar{x}_{10}$	$\bar{x}_{10}$	$\bar{x}_{10}$
BL_i (TE)	x_{30}	$\bar{x}_{30}$	$\bar{x}_{30}$		x_{30}
2	c_0	c_0	c_0	c_0	c_0
WL_1 (BE)	$\bar{x}_{20}$	$\bar{x}_{20}$	$\bar{x}_{20}$		$\bar{x}_{20}$
BL_i (TE)		1	0		
3	c_1	v_{40}	v_{70}	c_0	c_1
WL_1 (BE)		$\bar{x}_{30}$	$\bar{x}_{30}$		
BL_i (TE)	GND		V_{in}	V_{in}	
4	c_1	v_{50}	v_{80}	c_0	c_1
WL_1 (BE)					
BL_i (TE)	V_{in}	GND		V_{in}	
5	s'_0	v_{50}	v_{80}	c_0	c_1
WL_1 (BE)					
BL_i (TE)		V_r			
Verification	s'_0	s_0	v_{80}	c_0	c_1
WL_1 (BE)		GND			

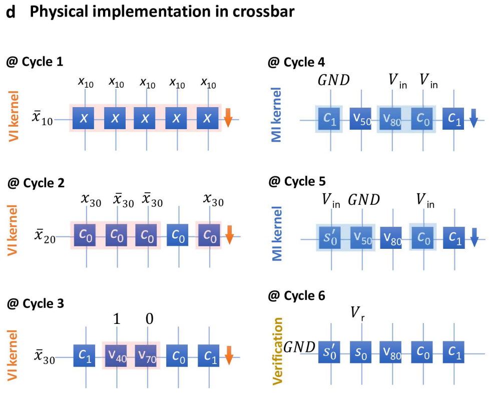

Fig. S6 Implementation of 1-bit full adder using (a) standard logic gates (NOR gates) in comparison to the design using (b) mixed-mode computing according to the control sequence shown in Fig. 3b. (c) Step-by-step control cycles for applying logic inputs through BLs and WLs in the crossbar configuration. (d) Physical implementation in memristor-based crossbar for each cycle.

Cycle	M11	M12	M13	M14	M21	M22	M23	M24	M31	M32	M33	M34	M41	M42	M43	M44	M45
1	c_0	c_0	c_0	c_0	c_0	c_0	c_0	c_0	c_0	c_0	c_0	c_0	c_0	c_0	c_0	c_0	c_0
2	c_1	v_{40}	v_{70}	c_0	c_1	c_1	c_1	c_0	c_1	c_1	c_1	c_0	c_1	c_1	c_1	c_0	c_1
3	c_1	v_{40}	v_{70}	c_0	c_2	v_{41}	v_{71}	c_0	c_2	c_2	c_2	c_0	c_2	c_2	c_2	c_0	c_2
4	c_1	v_{40}	v_{70}	c_0	c_2	v_{41}	v_{71}	c_0	c_3	v_{42}	v_{72}	c_0	c_3	c_3	c_3	c_0	c_3
5	c_1	v_{40}	v_{70}	c_0	c_2	v_{41}	v_{71}	c_0	c_3	v_{42}	v_{72}	c_0	c_4	v_{43}	v_{73}	c_0	c_4
6	c_1	v_{50}	v_{80}	c_0	c_2	v_{51}	v_{81}	c_0	c_3	v_{52}	v_{82}	c_0	c_4	v_{53}	v_{83}	c_0	c_4
7	s_0'	v_{50}	v_{80}	c_0	s_1'	v_{51}	v_{81}	c_0	s_2'	v_{52}	v_{82}	c_0	s_3'	v_{53}	v_{83}	c_0	c_4
8	s_0'	s_0	v_{80}	c_0	s_1'	s_1	v_{81}	c_0	s_2'	s_2	v_{82}	c_0	s_3'	s_3	v_{83}	c_0	c_4

Fig. S7 Implementation flow in memristive crossbar of 4-bit carry-ripple adder according to control sequence by M^{3}S demonstrated in Fig. 3b.

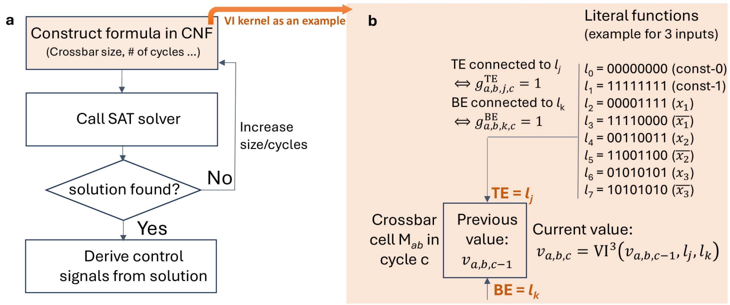

Fig. S8 (a) Flowchart illustrating the working principle of the $\mathrm{M^3S}$ automation tool for synthesizing and mapping logic circuits on memristive crossbars. The constructed Boolean formula, expressed in conjunctive normal form (CNF), serves as input to a Boolean satisfiability (SAT) solver to determine a solution. (b) Illustration of the V-mode operation in Eq. 1, providing an example of literal functions for $n = 3$ inputs. The table outlines the function table entries for each variable $l_{j,k}$ in the formula, demonstrating how the SAT solver assigns one variable for the top electrode (TE) and one for the bottom electrode (BE) to control which of the $(2n+2)$ literal functions is applied during each cycle. For each crossbar cell M_{ab} in cycle c, the Boolean satisfiability solver ensures that exactly one variable $g^{\mathrm{TE}}_{a,b,j,c}$ is set to 1 for the top electrode and exactly one variable $g^{\mathrm{BE}}_{a,b,j,c}$ is set to 1 for the bottom electrode, while all other variables are set to 0. This precise selection determines which of the $(2n+2)$ literal functions is applied during that cycle, enabling accurate control of the memristive logic operation.

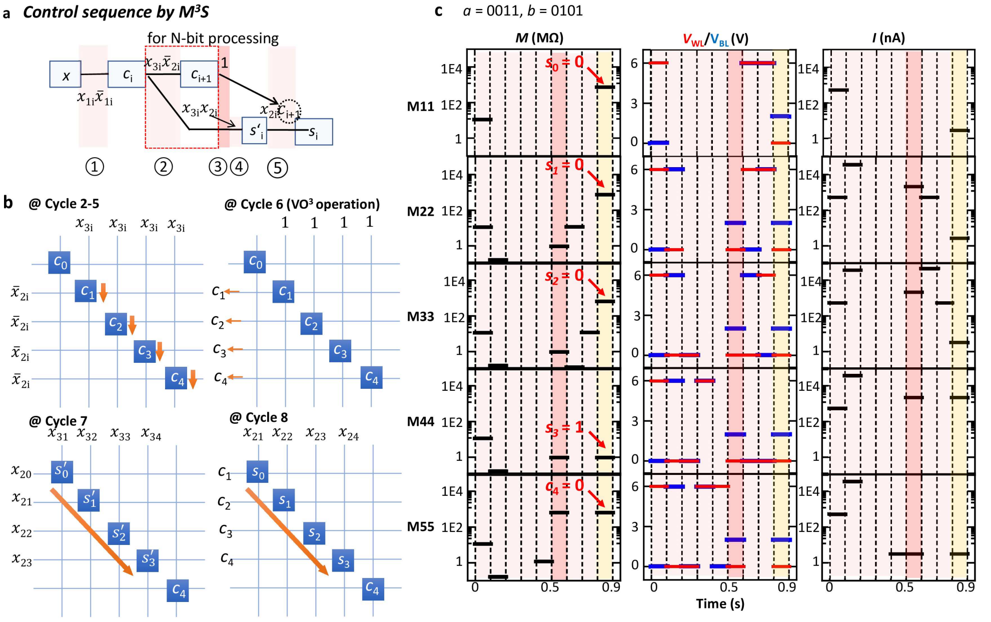

Fig. S9 Experimental implementation of N-bit carry-ripple adder by exploiting M^3S based on VI^3 and VO^3 . (a) Control sequence for N-bit carry-ripple adder by using $BiFeO_3$ memristive crossbar structure. (b) Demonstration of sequential operations for 4-bit carry-ripple adder with arbitrary inputs, which requires in total 13 cycles including four readout logic cycles (rLIV = 1, marked in dark red color). (c) Experimental results of 4-bit carry-ripple adder, i.e. memristance of each cell M, voltage on WL to shared bottom electrode V_{WL}, voltage on BL to individual top electrode V_{BL}, and current I from top electrode to bottom electrode across each cell.
